# Spectrum and Antibiotic Resistance of Uropathogens in Romanian Females

**DOI:** 10.3390/antibiotics9080472

**Published:** 2020-08-01

**Authors:** Răzvan-Cosmin Petca, Cristian Mareș, Aida Petca, Silvius Negoiță, Răzvan-Ionuț Popescu, Mihaela Boț, Enikő Barabás, Călin Bogdan Chibelean

**Affiliations:** 1 “Carol Davila”, University of Medicine and Pharmacy, 8 Eroii Sanitari blvd., 050474 Bucharest, Romania or razvan.petca@umfcd.ro (R.-C.P.); mihaela.bot@umfcd.ro (M.B.); 2 Department of Urology, “Prof. Dr. Th. Burghele” Clinical Hospital, 20 Panduri str., 050659 Bucharest, Romania; dr.marescristian@gmail.com (C.M.); dr.razvanp@gmail.com (R.-I.P.); 3 Department of Obstetrics and Gynecology, Elias University Hospital, 17 Marasti blvd., 011461 Bucharest, Romania; 4 Department of Anesthesiology and Critical Care, Elias University Hospital, 17 Marasti blvd., 011461 Bucharest, Romania; 5 George Emil Palade University of Medicine, Pharmacy, Science, and Technology of Targu-Mures, 38 Gheorghe Marinescu str., 540139 Targu-Mures, Romania; eniko.barabas@gmail.com (E.B.); calin.chibelean@umfst.ro (C.B.C.); 6 Department of Laboratory Medicine, Mureș County Hospital, 1st Gheorghe Marinescu str., 540136 Targu-Mures, Romania; 7 Department of Urology, Mureș County Hospital, 1st Gheorghe Marinescu str., 540136 Targu-Mures, Romania

**Keywords:** antibiotic resistance, AMR, urinary tract infections, uropathogens, *Escherichia coli*, female population, Romania, levofloxacin

## Abstract

Urinary tract infections (UTIs) in women represent a common bacteriological finding, with negligible recent and consistent research on antimicrobial resistance (AMR) in the female population. We designed a retrospective study to observe the incidence of frequent uropathogens and their resistance rates to common antibiotics. We elaborated multicenter research in three different teaching hospitals in Romania, analyzing 13,081 urine samples, of which 1588 met the criteria of inclusion. *Escherichia coli* (58.37%) was the most frequent Gram-negative uropathogen, presenting high resistance rates to levofloxacin (*R* = 29.66%), amoxicillin–clavulanic ac. (*R* = 14.13%), and ceftazidime (*R* = 6.68%). We found good sensitivity to imipenem and meropenem (both 98.16%), amikacin (*S* = 96.0%), and fosfomycin (*S* = 90.39%). The second most prevalent uropathogen was *Klebsiella* (16.93%), with the highest resistance quota to amoxicillin–clavulanic ac. (*R* = 28.62%), levofloxacin and nitrofurantoin (both *R* = 15.61%), and ceftazidime (*R* = 15.24%), and good sensitivity to imipenem (*S* = 93.93%), meropenem (*S* = 91.91%), and amikacin (*S* = 88.47%). *Enterococcus* (13.35%) was the most encountered Gram-positive pathogen. It proved the highest resistance to levofloxacin (*R* = 32.07%), penicillin (*R* = 32.07%), and ampicillin (*R* = 14.62%) and good sensitivity to vancomycin (*S* = 91.98%), fosfomycin (*S* = 94.4%), and nitrofurantoin (*S* = 89.15%). Considering the lack of recent and consistent data on this topic, we find our survey a valuable starting research study in this area with high significance for an accurate clinical approach.

## 1. Introduction

Urinary tract infections (UTIs) represent the most common bacterial infections in women and the second most common infectious presentation in community medical practice, after respiratory tract infections, accounting for significant morbidity and health care costs [1,2]. Studies suggest that each year over 150 million people worldwide are diagnosed with UTIs, affecting both genders of all age groups across their lifespan, usually requiring medical treatment [3]. Only in the United States, it accounts for more than eight million physician visits annually [4]. It seems that one in three women will experience an episode of UTI during her lifetime; one in five women repeats it in the same year [4,5]. The prevalence of UTIs among women increases with age, excepting young females, between 15 and 25 years old, which present a spike in the overall occurrence [6]. Over 20% of women older than 65 years old will experience an episode of UTI [7].

Apart from general considerations regarding risk factors for UTIs in both genders such as reduced fluid intake, delayed voiding, lithiasis of the urinary tract or chronic constipation, more specific risk factors in the general female population have been described such as anatomic conditions (short urethra) or age-related circumstances such as pregnancy, recent sexual intercourse, use of diaphragm or spermicides in young and adult women, or hormone-induced or anatomical modifications due to menopause in older women [4,7,8,9,10]. The factors contributing to the rising incidence in older women are their health status, presence of diabetes mellitus, history or current catheterization, residential status, history of antibiotic use, or spinal cord dysfunction [4,11].

UTIs are clinically categorized as complicated and uncomplicated. Uncomplicated infections generally occur only in female patients who have no neurological or structural abnormalities of the urinary tract and that are otherwise healthy individuals [12]. They are divided into lower urinary infections (cystitis) or upper urinary infections (pyelonephritis). Complicated UTIs are characterized by the association with factors that interfere with the host defense or compromised urinary tract, such as urinary obstruction, immunosuppression, renal transplantation, renal failure, retention caused by neurological disease, pregnancy or presence of foreign bodies such as indwelling catheters, calculi, or drainage devices [13]. Catheter-associated UTIs (CAUTIs) represent a class of high morbidity and mortality urinary infections, associated with specific risk factors, primarily female gender, besides diabetes and older age [14].

The etiology of UTIs and the antimicrobial sensitivity and resistance patterns vary significantly across different areas and in different periods [1,4,15]. Recent studies [16] suggest that *Escherichia coli* leads the etiology of UTIs among the European female population. Urease-producing bacteria such as *Klebsiella* spp., *Proteus* spp., and other Gram-negative pathogens, such as *Enterobacter* spp. and *Pseudomonas* spp., also play an essential role in this type of infection [13,17]. Gram-positive bacteria such as *Enterococcus* spp. or *Staphylococcus* spp. are the leading cause in nosocomial urinary infections as a consequence of selective pressure from the overusing of various antimicrobial agents in hospitalized patients [13,17]. Current data from Romania also suggest the leading of *E. coli* in the etiology of UTIs among females [18] and also males [19].

International research on female UTIs in European countries [20] has shown *E. coli* to have the highest resistance to ampicillin, trimethoprim/sulfamethoxazole, and nalidixic acid. They observed overall good sensitivity to fosfomycin and nitrofurantoin, followed by ciprofloxacin, amoxicillin/clavulanic ac, and cefuroxime. Considerable resistance to fluoroquinolones has been identified in Spain, Italy, and Russia. *Klebsiella* spp. has shown increasing resistance to cefuroxime, mecillinam, fosfomycin, and nitrofurantoin. *Proteus* spp. was less susceptible to non-beta-lactams and more susceptible to beta-lactams compared to other uropathogens. Except for ampicillin and trimethoprim/sulfamethoxazole, resistance to other agents was rare in *Staphylococcus* spp. Studies describe considerable intercountry variability for uropathogens’ resistance and sensitivity rates. Still, fosfomycin and mecillinam are the only agents that have preserved their susceptibility in female UTIs.

The early implementation by the World Health Organization of the Global Antimicrobial Resistance Surveillance System Report (GLASS) shows an improvement at a worldwide level with data reporting about all types of infections. In the GLASS report with data collected until May 2020, we can observe the increase in GLASS registered units in Europe with some 284 units in 2017, 1504 in 2018, and reaching a considerable number of 17,381 in 2019. However, the data collected are still reporting mainly bloodstream positive cultures, genital infections, and others; urinary infections are less documented. On the European continent, the GLASS report enrolled 25 countries, nine of them offered data for uropathogens, and only four (Finland, Latvia, Norway, and Switzerland) provided a complete description [21].

Insufficient data on this topic have led us to investigate the prevalence of UTIs among the Romanian female population in three different university hospitals. The main objective was to analyze urine culture results retrospectively and to determine the sensitivity and resistance rates of various uropathogens to common antibiotics and their drug-related resistance profiles. We appreciate this matter as an important and very real problem of public health worldwide.

## 2. Results

The survey included subjects from three different university hospitals in Romania: “Prof. Dr. Theodor Burghele” Clinical Hospital Bucharest (BCH), Elias University Hospital Bucharest (EUH), and Mures County Clinical Hospital (MCH). We identified a total number of 1588 female urine cultures to meet the criteria of inclusion in the study, of which 1343 (84.57%) had overall Gram-negative bacteria (BCH = 77.99%; MCH = 83.72%; EUH = 91.45%) and 245 (15.42%) had overall Gram-positive bacteria (BCH = 22.0%; MCH = 16.27%; EUH = 8.54%). *Escherichia coli* was the most common uropathogen in all three centers representing 927 (58.37%) of all bacteria; the highest incidence for *E. coli* was observed in EUH, where 444 (70.25%) of all urocultures were positive for this particular pathogen. Combined data from all centers, as well as the overall distribution of Gram-positive and Gram-negative bacteria incidence, are represented in Table 1.

In the female population, UTIs are characterized by an important incidence variation accordingly with sexual life dynamics, as well as with variability in hormone levels. In all centers, the highest incidence was observed in women over 55 years old, in menopause—1187 (74.47%), followed by young and sexually active women of 18–40 years old—206 (12.97%), and lastly by middle-aged women of 41–55 years old—195 (12.27%). Extensive results on age groups in all centers and overall statistics are represented in Table 2.

**Figure 1:**
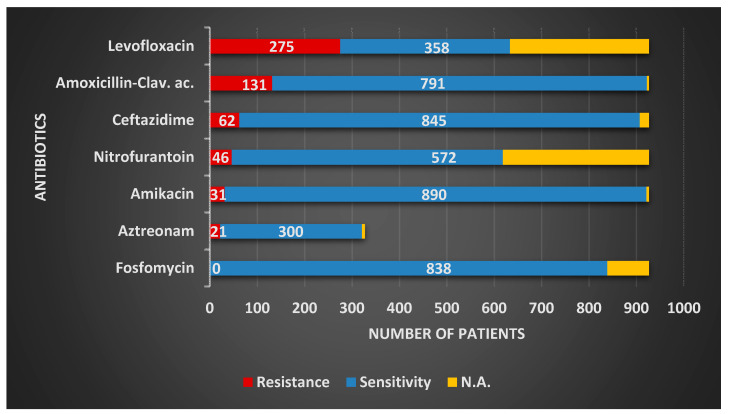
*E. coli* overall sensitivity and resistance profiles in the study group.

The most common Gram-negative pathogen, *E. coli*, presented the highest resistance to levofloxacin—29.66%, followed by amoxicillin–clavulanic ac.—14.13%, and ceftazidime—6.68%. A low resistance profile was determined for fosfomycin—0%, amikacin—3.34%, nitrofurantoin—4.96%, and carbapenems–imipenem—0% and meropenem—0.3%, respectively (Table 3 and Figure 1). For *Klebsiella* spp., the second most common Gram-negative pathogen, the highest resistance was observed for the combination of amoxicillin–clavulanic ac.—28.62%, followed by levofloxacin—15.61%. The lowest resistance for *Klebsiella* spp. was determined to carbapenems (imipenem—4.54%; meropenem—7.57%) and amikacin—11.52%. Extended results of resistance and sensitivity profiles for Gram-negative uropathogens are represented in Table 3.

The most common Gram-positive pathogen, *Enterococcus* spp., presented the highest resistance to levofloxacin and penicillin—32.07% and ampicillin—14.62%. A low resistance profile was determined for fosfomycin—0.62%, followed by vancomycin—1.41%, and nitrofurantoin—3.3%. *Staphylococcus* spp. presented high resistance to levofloxacin—27.27% and ceftazidime—22.72%. It showed low resistance to nitrofurantoin—0.8% and no resistance to fosfomycin. Expanded results of resistance and sensitivity profiles for Gram-positive uropathogens are represented in Table 4.

For Gram-negative pathogens, the highest overall resistance was observed for levofloxacin—26.58%, followed by amoxicillin–clavulanic ac.—18.16%, and nitrofurantoin—8.31%. The lowest resistance profile was seen for fosfomycin—2.03%, carbapenems (imipenem—2.18%; meropenem—3.52%), and amikacin—5.8%. For Gram-positive pathogens, the highest resistance was also observed for levofloxacin—31.42%, followed by penicillin—33.76%, and trimethoprim–sulfamethoxazole—15.0%. Low resistance patterns were found for fosfomycin—0.59%, followed by vancomycin—1.22%, and nitrofurantoin—3.79%. Detailed data analyses on drug-related resistance in conjunction with Gram-negative or Gram-positive characteristics of uropathogens are represented in Table 5.

## 3. Discussion

Considering the lack of recent and consistent data on uropathogen incidence among the female population, its stratification on various age-groups, the antimicrobial resistance (AMR) rates to common antibiotics, and the drug-related response patterns in relation to the Gram-negative or Gram-positive character of the confirmed pathogen, authors consider this paper as a reference in the regional bacteriological study. As current studies indicated increasing microbial resistance to standard treatments, empiric therapy recommendations that do not take local resistance into account can lead to inferior results. Local AMR patterns from an observational study over five months are provided, and their relation with the empiric treatment is discussed, considering those as key elements in female UTI management.

### 3.1. The Importance of Presenting Population Specific AMR Reports

The present study evaluated UTIs diagnosed in the female population from three different university hospitals in Romania. This determined, for the first time, the local AMR patterns for this region. The enrolled study group is a representative sample of the general population, correlating UTIs with age, presumed sexual activity, and menopausal status. Lately, according to worldwide trends, an increasing amount of studies focused their attention on bacterial agents and revealed increasing percentages in AMR, which were mostly caused by irrational usage. Improper empiric therapy for UTIs is usually identified as the most common cause of increasing AMR. The Romanian guidelines adapt information from the European Association of Urology (EAU) guidelines on UTIs, which are updated every year. Unfortunately, the lack of vast regional surveys in Eastern Europe on AMR, questions the applicability of their directives regarding empirical treatment in some populations.

The guideline states that antibiotics can be successfully used only if the local resistance is less than 20% [22,23]. The global tendency sustained by different studies in Europe and other continents showed increasing uropathogen AMR [19,24,25,26]. In contrast, there are some countries such as the Netherlands that always reported low resistance rates based mostly on rational antibiotic consumption [27].

Regardless of the Gram-positive or Gram-negative characteristics of pathogens, the highest AMR patterns were observed in levofloxacin, with an overall resistance of *R* = 27.32%. Bientinesi et al. [28] published this year extensive research of reports from the last ten years on the efficacy and safety of levofloxacin in the treatment of UTIs, raising the awareness of escalating resistance of this antimicrobial agent for all urinary pathogens. Other studies, such as ECO-SENS, that investigated the AMR of *E. coli* involved in acute uncomplicated UTIs in women to six common antibiotics, support decreasing susceptibility to fluoroquinolones. It comprised two surveys, ECO-SENS I in 2000 and -SENS II in 2008, and revealed an increasing resistance to ciprofloxacin in five European countries—Germany, Sweden, France, Spain, and the UK [24].

These results demonstrate similarities but also differences from other large European studies; thus, extensive territorial coverage with a significant sample population should be promoted.

### 3.2. Differences and Trends Regarding the Prevalence of Uropathogens

*Escherichia coli* has been demonstrated to be the most common uropathogen among the female population in the studied group, with an overall prevalence of 58.37%, with a higher incidence at EUH—70.25%, than BCH—52.91%, or MCH—46.15%. Similarities in frequency were also observed in UTIs involved in female patients, as other recent papers from European countries such as Italy—53.5% [29] and Hungary—42.5% [30] have previously reported. Higher percentages were obtained in the ARESC multicenter study, which involved nine European countries and Brazil. *E. coli* was identified as the leading agent in 76.7% of cases with significant variations between countries such as Austria (68.1%) and France (83.8%) [20]. In Eastern Europe, in countries such as Poland, a study conducted by Stefaniuk et al. in 2016 showed 75.6% for *E. coli* among the uropathogens [31].

The second most common uropathogen, *Klebsiella* spp., presented an overall incidence of 16.93%. The results show an increased rate of *Klebsiella* spp. in Romania compared to Northern Europe—8.2% or Southern Europe—9.4%, similar results to the Asian region—15.5%, and lower numbers compared to the American continent—28.8% [32].

*Enterococcus* spp. was the most common Gram-positive uropathogen and third in the overall incidence, its prevalence totaling 13.35%. The ARESC study [20] reported only 3% overall incidence of *Enterococcus* spp. in the studied countries.

Regarding *Proteus* spp., we encountered a 5.66% overall incidence. Extensive research by Schaffer [33] showed that this urease-producing pathogen causes nearly 4% of all UTIs. In the studied group, it was followed by *Pseudomonas* spp. with an overall prevalence of 3.58%. A study of 29 European countries conducted by Bouza et al. [34] observed similar results: *Proteus*—7.9%, followed by *Pseudomonas* spp.—6.9%., and *Staphylococcus* spp.—the least frequent uropathogen in the studied group—had 2.07% overall prevalence. A recent comprehensive review of the literature [35] showed that over 40% of young, sexually active females are colonized with *Staphylococcus* spp. in the urethra, cervix, or rectum at any given time. They serve as a major source of urinary inoculation from the gastrointestinal microbiota, representing a more common finding in women compared to male patients.

*Escherichia coli* is the leading bacterial agent responsible for urinary tract infections, even though its prevalence slightly differs over the world.

### 3.3. Antimicrobial Resistance in Relation to Patient Age

Considering age as a definitory risk factor for acquiring UTIs, we aimed to determine the variability of infectious status for particular age-groups in the female population. We linked the results with higher or lower sexual activity or postmenopausal status. The outcomes correlated the higher incidence of UTIs with postmenopausal status—74.74% (>55 years old). Multiple studies [36,37] admit various additional risk factors for older women considering hormonal (hypoestrogenism leading to genital atrophy), anatomic (uterine prolapse), or functional (urinary incontinence) modifications. Studies recommend local estrogen therapy as complementary in treating postmenopausal female UTIs [38]. A recent paper from Poland [39] that studied the incidence and resistance patterns of UTIs in menopausal women demonstrated the higher incidence among this particular age-group of female patients and observed higher rates of resistance to the tested antibiotics.

When analyzing *E. coli* prevalence based on age groups, the highest rate was determined after 55 years old. High percentages regarding bacterial incidence after 55 years were also obtained for the other analyzed uropathogens. Heytens et al. reported, in a 20 year survey performed in Belgium, an increased percentage of *E. coli* related UTIs in postmenopausal women compared to the younger population [40]. In a European study, the older population between 51–65 years was identified as a risk group for multidrug resistance urinary tract infections [41].

The second most vulnerable category of female patients acquiring UTIs in the studied group was the young and sexually active women—12.97% (18–40 years). A report from Italy found similar results for young and sexually active women—14.8% (19–45 years), but a slightly higher prevalence in the middle-aged group—19.4% (46–60 years); the comparable proportion for postmenopausal women was also suggested—47.4% (>61 years).

We documented that women getting older is the central basis for developing UTIs.

### 3.4. Comparison of Resistance Pattern for E. coli with Other Studies

*E. coli* was incriminating as the most common uropathogen among female patients in the studied group representing an overall of 58.37% of all strains. The highest resistance was observed to fluoroquinolones–levofloxacin (*R* = 29.66%). Resistance rates higher than 20% for fluoroquinolones were also reported in Poland and Russia in a study which included six European countries [41]. On the contrary, the same survey identified less than 20% resistance for the same antibiotic class in the German population. In the ARESC study, *E. coli* presented high susceptibility for fluoroquinolones such as ciprofloxacin, about 91.7%, and this result was not related to female hormonal status [20]. Seven years later, in 2016, Stephaniuk et al. observed in their study that AMR for ciprofloxacin raised to 24% for uncomplicated UTIs and over 41% for complicated UTIs [31]. Spain also reported increased resistance rates for quinolones [42].

We also report the following resistance patterns for amoxicillin–clavulanic ac.—*R* = 14.13%, ceftazidime—*R* = 6.68%, nitrofurantoin—*R* = 4.96%, amikacin—*R* = 3.34%, and no registered resistance to fosfomycin. In a Greek survey [43] that studied UTIs in female patients, similar results were observed for amoxicillin–clavulanic ac.—*R* = 5.9%, and for cephalosporins–cefaclor and cefprozil, *R* = 5.8% and *R* = 5.9%, respectively, and amikacin *R* = 2.0%. They observed considerably lower rates of resistance for fluoroquinolones–norfloxacin—5.5%. Another European study from Hungary [44] showed comparable results regarding *E. coli* resistance rates, as follows: amoxicillin–clavulanic ac.—*R* = 6%, cefuroxime—8%, nitrofurantoin—*R* = 2%, gentamicin—*R* = 7%, fosfomycin—0%.

An extensive recent European study [41] that aimed to determine the resistance patterns of *E. coli* in female UTIs also presented similar results to ours from various locations: Sweden: amoxicillin–clavulanic ac.—*R* = 8.9%, ceftazidime—*R* = 9.0%, nitrofurantoin—*R* = 0%, gentamicin—*R* = 7.7, fosfomycin—*R* = 2%; Germany: amoxicillin–clavulanic ac.—*R* = 32.9%, ceftazidime—*R* = 6.1%, nitrofurantoin—*R* = 0.4%, gentamicin—*R* = 3.9%, fosfomycin—*R* = 1.1%; Russia: amoxicillin–clavulanic ac.—*R* = 18.8%, ceftazidime—*R* = 13.7%, nitrofurantoin—*R* = 1.0%, gentamicin—*R* = 12.7%, fosfomycin—1.0%; very low resistance rates were observed in Finland, probably due to a more rigorous antibiotic policy nationwide, as follows: amoxicillin–clavulanic ac.—*R* = 0%, cefuroxime—*R* = 0%, nitrofurantoin—*R* = 0%, gentamicin—*R* = 3.3%, fosfomycin—*R* = 3.3%.

The increased AMR for *E. coli* should be a determinant cause for all physicians to reorganize their therapeutic strategies, relying as much as possible on the local resistance patterns, which should be continuously monitored.

### 3.5. Rationale for Reporting Gender-Specific Uropathogens AMR

We conducted a similar study on uropathogens in a Romanian male population, which gathered results from BCH and MCH, and was published in June 2020 [19]. Comparing the results, many similarities, but differences as well, were evident. As both surveys revealed, uropathogens displayed the same distribution pattern in male and female populations, persons older than 55 years presented an increased risk of developing UTIs, even though the causes were mostly different. *E. Coli* occupied the leading position, followed by *Klebsiella* spp., and *Proteus* spp. for Gram-negative and *Enterococcus* spp. as the main identified bacteria for Gram-positive. In female subjects, *E. Coli* was isolated in 58.37% of Gram-negative bacteria, a higher number compared to 35.98% isolated in males. *Klebsiella* spp. and *Proteus* spp. were found in almost equal percentages in both groups.

For fluoroquinolones, represented by levofloxacin, resistance rates were as follows for females vs. males: *E. coli*—29.66% vs. 37.69%, *Klebsiella* spp.—15.61% vs. 45.36%, *Enterococcus* spp. 32.07% vs. 42.04%. Analyzing amoxicillin–clavulanic ac., we observed *E. coli* resistance in 14.13% vs. 28.03%, and *Klebsiella* spp. resistance in 28.62% vs. 65.58%, for females vs. males. Scrutinizing data from *Proteus* spp., the resistance pattern showed almost no differences between the male and female populations. *Enterococcus* spp. also showed increased rates of resistance for penicillin (32.07% vs. 33.52%) and ampicillin (14.62% vs. 15.9%) in females vs males. Increased sensitivity rates were obtained for fosfomycin, nitrofurantoin, and vancomycin in both groups.

The staggering resistance rates of the most common Gram-negative strains in the male population, compared to a similar-sized group of female subjects, stands out. This must be related to the more prevalent complicated infections in males.

These data demonstrate the need for sex-segregated surveys to cover larger territories, providing a robust base for the international guidelines, supporting the every-day practice for all physicians.

### 3.6. Empiric Antibiotic Treatment in Female UTIs

UTIs represent a common cause of medical presentation, with an average global cost per year in the USA of nearly USD 4 billion [4]. Its diagnosis relies on various clinical and paraclinical investigations, of which the uroculture is often mandatory. The European Association of Urology (EAU) [22] recommends empiric treatment for a single-case scenario when uroculture is not necessary. The first episode, uncomplicated cystitis, represents the vast majority of UTIs in women. Still, initially, all UTI cases rely on empiric treatment until the uroculture is performed, thus emerging the necessity of knowing the local incidence of common uropathogens and their resistance patterns. The overall resistance for all pathogens was the lowest for fosfomycin—*R* = 1.82%, followed by imipenem—*R* = 2.18%, meropenem—*R* = 3.52%, amikacin—*R* = 5.97%, nitrofurantoin—*R* = 7.6%, and ceftazidime—*R* = 9.7%. An increasing resistance was observed for ampicillin—*R* = 14.62%, amoxicillin–clavulanic ac.—*R* = 18.16%, and trimethoprim–sulfamethoxazole—*R* = 15.0%; a very high resistance was determined for levofloxacin—*R* = 27.32% and penicillin—*R* = 33.76%.

In the Romanian population, most antibiotics used to treat uncomplicated urinary bacterial diseases are generally empirically prescribed, mostly according to the European guidelines. The lack of data for local bacterial prevalence and antibiotic susceptibility often creates a difficult situation for practitioners in prescribing other drugs and may become crucial for increasing bacterial resistance. The nowadays trend adjusts routine therapy as the selection of antibiotics according to bacterial patterns for a specific region.

The EAU [22] and The American Urology Association (AUA) [23] recommend certain antimicrobials as empiric treatment for uncomplicated UTIs, as long as the reported local resistance patterns do not exceed 20% resistance. Nitrofurantoin and fosfomycin fit international recommendations for local resistance patterns. In upper urinary tract infections, cephalosporins, fluoroquinolones, and aminoglycosides are recommended. Carbapenems are indicated only in patients positive for multidrug-resistant pathogens. Guideline recommendations imply the use of fluoroquinolones only if local resistance is under 10%. The European Commission implemented restrictions in March 2019 regarding the use of fluoroquinolones considering their resistance and long-lasting effects that apply in all EU countries [45].

Optimal empirical therapy of urinary tract infection requires accurate knowledge of local susceptibility patterns, which may vary with the organism and patient characteristics. The current study affirms that for the Romanian female population, usage of fluoroquinolones is no longer suitable in treating urinary infections as far as its resistance exceeds 20%. Ceftazidime, amikacin, and carbapenems are still reliable options.

### 3.7. Limitations

This study had certain limitations. Some antibiotics (e.g., norfloxacin, ofloxacin, gentamicin, ceftriaxone, cefotaxime, pivmecillinam), although valuable therapeutic options, are not routinely tested by the hospital’s microbiology laboratory; thus, they are not included in our results. As long as trimethoprim/sulfamethoxazole is not regularly tested for Gram-negative bacteria, this study cannot correlate one of the first line European therapeutic options in treating uncomplicated UTIs.

The retrospective character of the study seriously influences information regarding risk factors, underlying diseases, preoperative or postoperative status, associated medication, previous antibiotic administration, recurrences, and lifestyle. The authors could not have determined complex correlations between these factors for a better understanding of sophisticated resistance mechanisms.

We conducted our study in five months during the fall–winter season, when the virulence of uropathogens is increased as well as the prevalence of UTIs in the general population. We consider that a more extended period of observation could more accurately reproduce the real distribution and resistance patterns of UTIs in the female population.

The strength of is this study is the multicenter coverage on the female population at different ages in two separate Romanian regions because the discussed topics correlate the actual guideline recommendations with the local susceptibility pattern. As the first study of this kind for this country, the data presented may be considered as an option to adjust the general European recommendations for antibiotic usage in treating urological infections in women according to presented bacterial patterns.

## 4. Materials and Methods

### 4.1. Study Design and Sample Population

We designed a multicenter retrospective study in three different tertiary centers in Romania: “Prof. Dr. Theodor Burghele” Clinical Hospital Bucharest (BCH), Elias University Hospital Bucharest (EUH), and Mures County Clinical Hospital (MCH). We gathered results from female patients registered between 1 September 2018 and 31 January 2019. The study was conducted in accordance with the Declaration of Helsinki. The data collected retrospectively did not contain any personal information. For each patient, written informed consent was obtained. The Ethics Committee from every hospital approved the protocol: Burghele Clinical Hospital (no.2/2019), Mures County Hospital (no. 6522/2020), and Elias University Hospital (no. 2517/2020).

**Figure 2:**
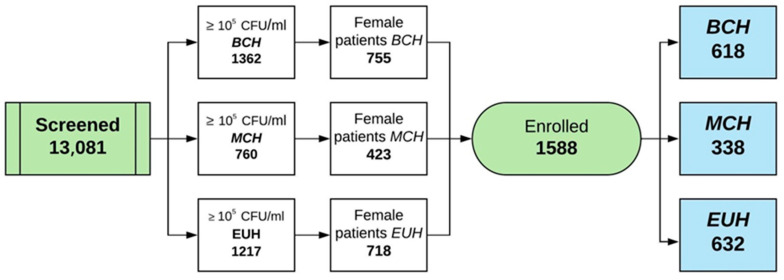
Diagram of screened and enrolled patients in the study.

A total number of 13,081 midstream urine samples from all three centers were analyzed for bacterial determination through standard urine cultures, of which 3339, both males and females, presented more than 10^5^ CFU/mL. We determined 1896 female urine cultures that showed over 10^5^ CFU/mL, of which 1588 met the criteria of inclusion. The representative diagram of patient dynamics is illustrated in Figure 2.

In all three centers, the enrolled patients were given ambulatory care and hospitalized. Thus, an extensive background regarding a more complex medical history for ambulatory patients could not be provided; various demographic information such as the age, sex, or social status of each patient was recorded.

### 4.2. Inclusion and Exclusion Criteria

The inclusion criteria:

1. Positive uroculture ≥10^5^ CFU/mL;
2. Single bacteria strain on uroculture;
3. Female patients;
4. Age ≥18 years.

The exclusion criteria:

1. Uroculture <10^5^ CFU/mL;
2. Multiple bacteria strain on uroculture;
3. Presence of a urological catheter.

### 4.3. Antibiotics Policy in UTIs in Study Centers

We applied a prudent policy for the administration of antibiotics in the treatment of UTIs in all three centers, according to both Romanian and European Associations of Urology [22,46] on Urinary Infections Guidelines, which are continually updating. Standard courses of antibiotics considering the level of infection were provided, as various types of UTIs imply different classes of antibiotics and distinct regimes. Uncomplicated cystitis is a relatively frequent finding in female patients. A single dose of fosfomycin or a small dosage of nitrofurantoin or trimethoprim–sulfamethoxazole were administered. In upper UTIs, such as pyelonephritis, a cephalosporin, aminoglycoside, or fluoroquinolone was the drug of choice considering the extended-spectrum of action and the better tissue diffusion to penetrate the kidney parenchyma. In patients with multidrug resistance uropathogens or systemic septic status admitted to intensive care units, new generations of carbapenems were administered with or without an aminoglycoside. Whenever Gram-positive bacteria were suspected, various combinations of ampicillin, penicillin, or vancomycin were employed. In all cases, an empiric course of antibiotics was initiated at the presentation considering the associated pathology in conjunction with available guidelines, followed by a targeted treatment on bacterial resistance, and a sensitivity profile as soon as the antibiogram was available.

Examining urine for bacterial load implies a minimum of seven days time-lapse between testing and the last antibiotic treatment in all centers.

### 4.4. Sample Collection, Bacterial Culture, and Identification

In all cases, the urine collecting technique was conducted complying with all International Safety Standards [47]. The urine was collected in a sterile receptacle followed by culture on lactose agar and Columbia sheep agar. For various microorganisms such as *Staphylococcus* spp., a different culturing medium such as Chapman was used. All media used were produced by BioMerieux^®^, purchased ready-to-use on Petri dishes in BCH and EUH. In MCH, inhouse made culture media were used, followed by internal quality control in all centers. After 24 h of incubation time at around 37 °C, we identified the microorganisms based on the specific morphology, gram reactions, and biochemical characteristics of each pathogen agent. We considered as positive for our research only cultures that presented more than 10^5^ CFU/mL, as well as pure cultures. Manual technique and the Vitek 2 automatic microbiology system were utilized for density determination—CFU/mL [48,49]. Consecutively to the Petri dish inoculation of urine specimen, for the manual technique, the bacteriuria was calculated according to formula X = N × D × 1/inoculated volume, where X = number of CFU/mL, N = number of colonies on Petri dish, and D = dilution factor (inverse of dilution). The results are filed following the order of magnification of bacteriuria, e.g., <10^3^, 10^3–4^, 10^4–5^, and ≥10^5^ CFU/mL [48].

The bacteria were identified at species level in most of the cases by phenotype. Microorganism determination was conducted based on various morphology characteristics, biochemical properties—lactose positivity or negativity, urease, indole, lysine-decarboxylase, or H_2_S production for enterobacteria. Regarding morphology characteristics, for the majority of enterobacteria, Gram-staining determined Gram-negative-bacilli or coccobacilli for *Klebsiella* spp. A key element of *Staphylococcus* spp. characteristics is represented by catalase-positivity; they are Gram-positive cocci, growing in a Chapman medium. *Staphylococcus aureus* presents as yellow cocci colonies. In Columbia sheep agar, beta-hemolysis and pigment production are definitory. To identify *Pseudomonas* spp. strains, colony-morphologies (beta hemolysis, pigment production, pleasant smell colonies with metallic shine), oxidase-positivity, Gram-negative rods, or non-fermenting properties were analyzed. Enterococci are Gram-positive bacteria that present as small colonies, characterized by catalase-negativity and alpha-hemolysis in an agar–bile–esculin medium with positive properties. For uncertain positive diagnosis at particular levels, we used the Vitek 2 automatic system [49].

### 4.5. Antibiotic Susceptibility Test

We followed the Clinical Laboratory Standards Institute (CLSI) guidelines [50] for the disk diffusion technique for the antibiogram we used to determine the antimicrobial susceptibility for each bacterial strain. After we swabbed a standard inoculum adjusted to 0.5 McFarland in the Petri dish, no more than six disks were used on a standard size of 90 mm. Whenever seven or more antibiotics were tested, a 150 mm plate was utilized, so each zone diameter was clearly measurable, as overlapping zones hinder accurate evaluation; we placed eight antibiotic discs at an equal distance from each other and the same distance from the border of the dish and the ninth antibiotic disc in the center of the plate. After another 18–24 h of incubation, we measured the distance between the antibiotic disc and the bacterial inoculum according to the disk diffusion technique (Kirby–Bauer) and compared our results with CLSI guidelines from 2018, to determine the resistance and the susceptibility of each pathogen agent.

The concentration of each antimicrobial agent disc we tested and the length of each intermediate-inhibition zone (in mm) for *E. coli*, the most frequent uropathogen, as well other members of the *Enterobacteriaceae* family (*Klebsiella* spp. and *Proteus* spp.), according to CLSI guidelines [50], are represented in Table 6; values higher than the upper limit represent the criterion of sensibility, while values lower than the inferior limit represent the criterion of resistance.

### 4.6. Statistical Analysis

Data obtained were analyzed using Microsoft Excel software (version 2016, Microsoft Corporation, Redmond, WA, USA), and simple descriptive statistics were calculated. The relation of variables was analyzed using the frequency and percentage.

## 5. Conclusions

Women with clinical manifesting UTIs that urge physicians to prescribe antibiotics should never be treated empirically with levofloxacin. Concerning the low resistance to nitrofurantoin and fosfomycin, they are appropriate to be prescribed for the empirical treatment of uncomplicated UTIs.

## Figures and Tables

**Table 1:** Isolated uropathogens in the study group.

Isolated Bacteria	BCH		MCH		EUH		Total	
	*n*	*%*	*n*	*%*	*n*	*%*	*n*	*%*
Gram Negative	482	77.99	283	83.72	578	91.45	1343	84.57
*Escherichia coli*	327	52.91	156	46.15	444	70.25	927	58.37
*Klebsiella* spp.	95	15.37	71	21.05	103	16.29	269	16.93
*Pseudomonas aeruginosa*	14	2.26	32	9.46	11	1.74	57	3.58
*Proteus* spp.	46	7.44	24	7.1	20	3.16	90	5.66
Gram Positive	136	22.0	55	16.27	54	8.54	245	15.42
*Enterococcus* spp.	114	18.44	47	13.9	51	8.06	212	13.35
*Staphylococcus* spp.	22	3.55	8	2.36	3	0.47	33	2.07

*n*—number, *%*—percentage, BCH—Burghele Clinical Hospital, MCH—Mures County Hospital, EUH—Elias University Hospital.

**Table 2:** Uropathogens in female patients of various age groups in the study population

Isolated Bacteria	BCH						MCH						EUH						Total					
	≤40		41–55		>55		≤40		41–55		>55		≤40		41–55		>55		≤40		41–55		>55	
	*n*	*%*	*n*	*%*	*n*	*%*	*n*	*%*	*n*	*%*	*n*	*%*	*n*	*%*	*n*	*%*	*n*	*%*	*n*	*%*	*n*	*%*	*n*	*%*
*Escherichia coli*	51	8.25	48	7.76	228	36.89	22	6.5	23	6.8	111	32.84	49	7.75	31	4.9	364	57.59	122	7.68	102	6.42	703	44.26
*Klebsiella* spp.	13	2.1	14	2.26	68	11.0	8	2.36	13	3.84	50	14.79	7	1.1	6	0.94	90	14.24	28	1.76	33	2.07	208	13.09
*Pseudomonas aeruginosa*	2	0.32	1	0.16	11	1.77	2	0.59	3	0.88	27	7.98	-	-	3	0.47	8	1.26	4	0.25	7	0.44	46	2.89
*Proteus* spp.	7	1.13	7	1.13	32	5.17	5	1.47	4	1.18	15	4.43	-	-	2	0.31	18	2.84	12	0.75	13	0.81	65	4.09
*Enterococcus* spp.	19	3.07	23	3.72	72	11.65	9	2.66	7	2.07	31	9.17	6	0.94	2	0.31	43	6.8	34	2.14	32	2.01	146	9.19
*Staphylococcus* spp.	4	0.64	7	1.13	11	1.77	-	-	1	0.29	7	2.07	2	0.31	-	-	1	0.15	6	0.37	8	0.5	19	1.19
Total	96	15.53	100	16.18	422	68.28	46	13.6	51	15.08	241	71.3	64	6.96	44	6.96	524	82.91	206	12.97	195	12.27	1187	74.74

*n*—number, ***%***—percentage, BCH—Burghele Clinical Hospital, MCH—Mures County Hospital, EUH—Elias University Hospital.

**Table 3:** Overall antibiotic resistance profile in Gram-negative uropathogens.

Antibiotics	Gram-Negative Organism Isolated																			
	*Escherichia coli*					*Klebsiella* spp.					*Pseudomonas aeruginosa*					*Proteus* spp.				
	R		S		NA	R		S		NA	R		S		NA	R		S		NA
	*n*	*%*	*n*	*%*		*n*	*%*	*n*	*%*		*n*	*%*	*n*	*%*		*n*	*%*	*n*	*%*	
Amikacin	31	3.34	890	96.0	-	31	11.52	238	88.47	-	8	14.03	46	80.7	-	8	8.88	77	85.55	-
Amoxicillin-Clavulanic ac.	131	14.13	791	85.32	-	77	28.62	199	73.97	-	12	21.05	21	36.84	-	24	26.66	63	70.0	-
Aztreonam	21	6.42	300	91.74	600(M + E)	20	21.05	71	74.73	174(M + E)	3	21.42	11	78.57	43(M + E)	4	8.69	41	89.13	44(M + E)
Ceftazidime	62	6.68	845	91.15	-	41	15.24	220	81.78	-	14	24.56	41	71.92	-	13	14.44	76	84.44	-
Fosfomycin	0	0	838	90.39		-	-	-	-	-	4	12.5	15	46.87	25(B + E)	16	66.66	5	20.83	66(B+E)
Imipenem	0	0	321	98.16	600(M + E)	9	4.54	186	93.93	71(M)	4	16.0	20	80.0	32(M)	0	0	45	97.82	44(M + E)
Levofloxacin	275	29.66	358	38.61	-	42	15.61	224	83.27		18	31.57	38	66.66	-	22	24.44	63	70.0	-
Meropenem	1	0.3	321	98.16	600(M + E)	15	7.57	182	91.91	71(M)	4	16.9	20	80.0	32(M)	1	2.17	42	91.32	44(M + E)
Nitrofurantoin	46	4.96	572	61.7	-	42	15.61	113	42.0	-	8	18.6	26	60.46	14(B)	9	37.5	11	45.83	66(B + E)

*n*—number, ***%***—percentage; **R**—resistant, **S**—sensitive, **NA**—not applicable; B—BCH—Burghele Clinical Hospital; M—MCH—Mures County Hospital; E—EUH—Elias University Hospital.

**Table 4:** Gram-positive uropathogens resistance profile.

Antibiotics	Gram-Positive Organism Isolated									
	*Enterococcus* spp.					*Staphylococcus* spp.				
	R		S		NA	R		S		NA
	*n*	*%*	*n*	*%*		*n*	*%*	*n*	*%*	
Amikacin	5	10.63	15	31.91	165(B + E)	2	6.06	30	90.9	-
Ampicillin	31	14.62	166	78.3	-	-	-	-	-	-
Trimethoprim–Sulfamethoxazole	5	10.63	18	38.29	165(B + E)	7	21.21	21	63.63	-
Ceftazidime	2	4.25	19	40.42	165(B + E)	5	22.72	17	77.27	11(M + E)
Fosfomycin	1	0.62	152	94.4	51(E)	0	0	5	62.5	25(B + E)
Levofloxacin	68	32.07	141	66.5	-	9	27.27	21	63.63	-
Nitrofurantoin	7	3.3	189	89.15	-	2	0.8	19	76.0	8(M)
Penicillin	68	32.07	129	60.84	-	11	50.0	10	45.45	11(M + E)
Vancomycin	3	1.41	195	91.98	-	-	-	-	-	-

*n*—number, ***%***—percentage; R—resistant, S—sensitive, NA—not applicable; B—BCH—Burghele Clinical Hospital; M—MCH—Mures County Hospital; E—EUH—Elias University Hospital.

**Table 5:** Gram-negative and Gram-positive uropathogens’ overall resistance to common antibiotics.

Antibiotics	Gram-Negative				Gram-Positive				Total			
	R		S		R		S		R		S	
	*n*	*%*	*n*	*%*	*n*	*%*	*n*	*%*	*n*	*%*	*n*	*%*
Amikacin	78	5.8	1251	93.14	7	8.75	45	56.25	85	5.97	1296	91.07
Amoxicillin–Clavulanic ac.	244	18.16	1074	79.97	-	-	-	-	244	18.16	1074	79.97
Ampicillin	-	-	-	-	31	14.62	166	78.3	31	14.62	166	78.3
Aztreonam	48	9.95	423	87.75	-	-	-	-	48	9.95	423	87.75
Trimethoprim-Sulfamethoxazole	-	-	-	-	12	15.0	39	48.75	12	15.0	39	48.75
Ceftazidime	130	9.67	1182	88.01	7	10.14	36	52.17	137	9.7	1218	86.26
Fosfomycin	20	2.03	858	87.28	1	0.59	157	92.89	21	1.82	1015	88.10
Imipenem	13	2.18	572	95.97	-	-	-	-	13	2.18	572	95.97
Levofloxacin	357	26.58	683	50.85	77	31.42	162	66.12	434	27.32	845	53.21
Meropenem	21	3.52	565	94.79	-	-	-	-	21	3.52	565	94.79
Nitrofurantoin	105	8.31	722	57.16	9	3.79	208	87.76	114	7.6	930	62.0
Penicillin	-	-	-	-	79	33.76	139	59.4	79	33.76	139	59.40
Vancomycin	-	-	-	-	3	1.22	195	79.59	3	1.22	195	79.59

*n*—number, *%*—percentage; R—resistant, S—sensitive.

**Table 6:** Zone diameter interpretive standards chart for the Disk Diffusion method for the *Enterobacteriaceae* family [50].

Antibiotics	Quantity (mcG)	Disk Diffusion Ranges (mm)
Amikacin	30	15–16
Amoxicillin–Clavulanic ac.	20/10	14–17
Aztreonam	30	18–20
Ceftazidime	30	18–20
Fosfomycin	200	13–15
Imipenem	10	20–22
Levofloxacin	5	14–16
Meropenem	10	20–22
Nitrofurantoin	300	15–16

S—values higher than the upper limit, R—values lower than the inferior limit.
